# Clinical validation of an evidence-based method to adjust Pancreatic Enzyme Replacement Therapy through a prospective interventional study in paediatric patients with Cystic Fibrosis

**DOI:** 10.1371/journal.pone.0213216

**Published:** 2019-03-12

**Authors:** Joaquim Calvo-Lerma, Jessie Hulst, Mieke Boon, Carla Colombo, Etna Masip, Mar Ruperto, Victoria Fornés-Ferrer, Els van der Wiel, Ine Claes, Maria Garriga, Maria Roca, Paula Crespo-Escobar, Anna Bulfamante, Sandra Woodcock, Sandra Martínez-Barona, Ana Andrés, Kris de Boeck, Carmen Ribes-Koninckx, on behalf of MyCyFAPP project

**Affiliations:** 1 Instituto de Investigación Sanitaria La Fe de Valencia, Valencia, Spain; 2 Universitat Politècnica de València, Research Institute of Food Engineering for Development, Valencia, Spain; 3 Erasmus Medical Center, Sophia Children’s Hospital, GD Rotterdam, the Netherlands; 4 Pediatric Pulmonology and Cystic Fibrosis Unit, Department of Pediatrics, University Hospitals Leuven, Leuven, Belgium; 5 CF Center, Università degli Studi di Milano, Fondazione IRCCS Ca’ Granda, Ospedale Maggiore Policlinico, Via Commenda 9, Milan, Italy; 6 Hospital Universitario Ramón y Cajal, Carretera Colmenar Viejo km 9, Madrid, Spain; National Institute for Agronomic Research, FRANCE

## Abstract

**Background:**

A method to adjust Pancreatic Enzyme Replacement Therapy in Cystic Fibrosis is not currently available.

**Objectives:**

To assess the *in vivo* efficacy of a method to adjust the dose of enzymatic supplement in CF extrapolated from previous *in vitro* digestion studies (theoretical optimal dose, TOD). Secondly, to assess how individual patient characteristics influence the expected coefficient of fat absorption (CFA) and thus to identify an individual correction factor to improve TOD.

**Methods:**

A prospective interventional study in 43 paediatric patients with CF from 5 European centres. They followed a 24h fixed diet with the theoretical optimal dose for each meal. Faecal collection was carried out between colorimetric markers in order to include all the faeces corresponding to the fixed diet. Beta regression models were applied to assess the associations of individual patient characteristics with the CFA.

**Results:**

Median CFA was 90% (84, 94% 1^st^, 3^rd^ Q.) with no significant differences among centres. Intestinal transit time was positively associated with CFA (p = 0.007), but no statistical associations were found with and age, gender, phenotype or BMI. Regression model showed no improvement of the *in vitro* predicted theoretical optimal dose when taking individual patient characteristics into account.

**Conclusion:**

Strict adherence to the theoretical optimal dose of enzymatic supplement for a prescribed meal, led to median CFA levels at the clinical target of 90% with a low variability between patients. The proposed method can be considered as a first approach for an evidence-based method in PERT dosing based on food characteristics. Results have to be confirmed in free dietary settings.

## Introduction

Pancreatic insufficiency is associated with Cystic Fibrosis (CF) in up to 85–90% of the patients. Pancreatic Enzyme Replacement Therapy (PERT) is the indicated treatment to compensate for maldigestion and malabsorption of nutrients [1], and aims at maintaining or achieving an adequate nutritional status. In the 80s, enteric-coated pancrelipase enzyme supplements were applied in the clinical practice for the first time, showing an improvement in faecal fat losses as compared to the conventional pancreatic enzymes [2,3]. This enteric-coated formulation became from then onwards the standard treatment for PERT in CF. However, despite PERT, fat absorption remains insufficient in most of the patients with CF, and the inaccuracy in the dosing criterion has been suggested as a possible reason for this [4]. Over the last decades, several studies have concluded that evidence-based guidance is needed to adjust the dose of enzymatic supplements given in PERT. However, to our knowledge, no successful attempt has been made up to date [5–7].

Determination of fat in faeces and calculation of the coefficient of fat absorption (CFA) have been used as the gold standard method to assess enzymatic supplement dosage efficacy [6]. The CFA reflects the amount of fat excreted in faeces in relation to the dietary intake of fat. Recently, an enormous variability in the response to doses used in PERT among patients has been confirmed, with no clear association between CFA and the dose of the enzymatic supplement [8,9]. Moreover, wide intra-and inter- patient ranges of the dose of enzymatic supplement (LU/g fat) in a multicentre observational study were showed [10].

Recent research has identified the important influence of foods’ characteristics on the process of lipolysis [7, 11–13]. Few investigations have considered integration of the specific food characteristics of a wide range of foods to estimate the dose of the enzymatic supplement and no trials have been performed to study this *in vivo*.

The main goal of the Horizon 2020 MyCyFAPP Project is to establish an evidence-based method to identify the optimal dose of enzymatic supplements in CF, which will allow for patients’ self-management of this therapy by means of a mobile app [14]. This project included an *in vitro* digestion study of foods under standard CF simulated gastrointestinal conditions in order to determine the theoretical optimal dose of enzymatic supplement (TOD) per food product, based on food characteristics. In this study, the modelling of results confirmed that not only the amount of fat, but also the food characteristics–e.g. amount of other nutrients or matrix properties–led to very significant variation in the doses of the enzymatic supplement requirement for optimal digestion of food products with the same total amount of fat.

Next, the project aims to evaluate the efficacy of the TODs of the enzymatic supplement when applied *in vivo* to patients. We hypothesized that an individual correction factor would be identified for each patient to correct for individual patient characteristics (gender, age, mutation type, transit time). Correction of the TOD by an individual correction factor may result in the individual optimal dose, leading to the optimal dose of enzymatic supplement needed to obtain an optimal CFA *in vivo*.

Therefore, the first aim of the present study was to assess the *in vivo* efficacy of an evidence-based prediction model to adjust the dose of the enzymatic supplements used in PERT obtained by means of an *in vitro* digestion experimental setting. Secondly, we aimed at identifying an individual correction factor, based on individual patient characteristics, representing the difference between the *in vitro* and *in vivo* scenarios. Therefore, in a prospective interventional pilot study, we evaluated the variability in CFA between patients after consumption of a predefined 24 hours diet in combination with the TOD of enzyme supplements, to identify this individual correction factor.

## Materials and methods

### Subjects

Paediatric patients with CF followed up as outpatients in 5 European CF centres participating in the MyCyFAPP project: Madrid (Spain), Valencia (Spain), Milan (Italy), Leuven (Belgium) and Rotterdam (The Netherlands) were considered for enrolment.

Inclusion criteria required a confirmed diagnosis of CF (a documented sweat chloride ≥ 60 mEq/L and a documented genotype with two disease-causing mutations in the CFTR gene), age between 2 and 18 years, having pancreatic insufficiency (faecal elastase < 200 mcg/g faeces), and need for treatment with PERT, having a stable clinical status at least two weeks before signing the informed consent and patients’ having the capacity and willingness to fulfil the test meals and the faeces collection. Patients were considered not stable to participate if they would meet any of the exclusion criteria as explained below. CFA prior the inclusion in the study was disregarded, as the aim was not to measure relative intra-patient improvement of this parameter but to assess the CFA result achieved with the proposed dose optimisation method.

The exclusion criteria were the presence of an acute infection associated with decreased appetite or fever at the time of the run-in visit, acute abdominal pain, severe liver disease in the assumption that in a few patients it may be associated with a certain degree of cholestasis, FEV_1_ <40% predicted, severe hypoalbuminemia (<2.5g/ml in blood), hospitalisation admissions or intravenous antibiotics <2 weeks before signing the informed consent, changes in the usual treatment (prokinetics, proton pump inhibitors, H_2_ blockers and antibiotics) <2 weeks before signing the informed consent, presence of other alterations that would potentially endanger the safety of the patient as estimated by the researchers (for example other drugs, newly diagnosed CF-related diabetes, etc.) and hypersensitivity or adverse reactions to the enzymatic supplements. Lung transplantation was considered an exclusion criterion too.

### Study design

**Fig 1:**
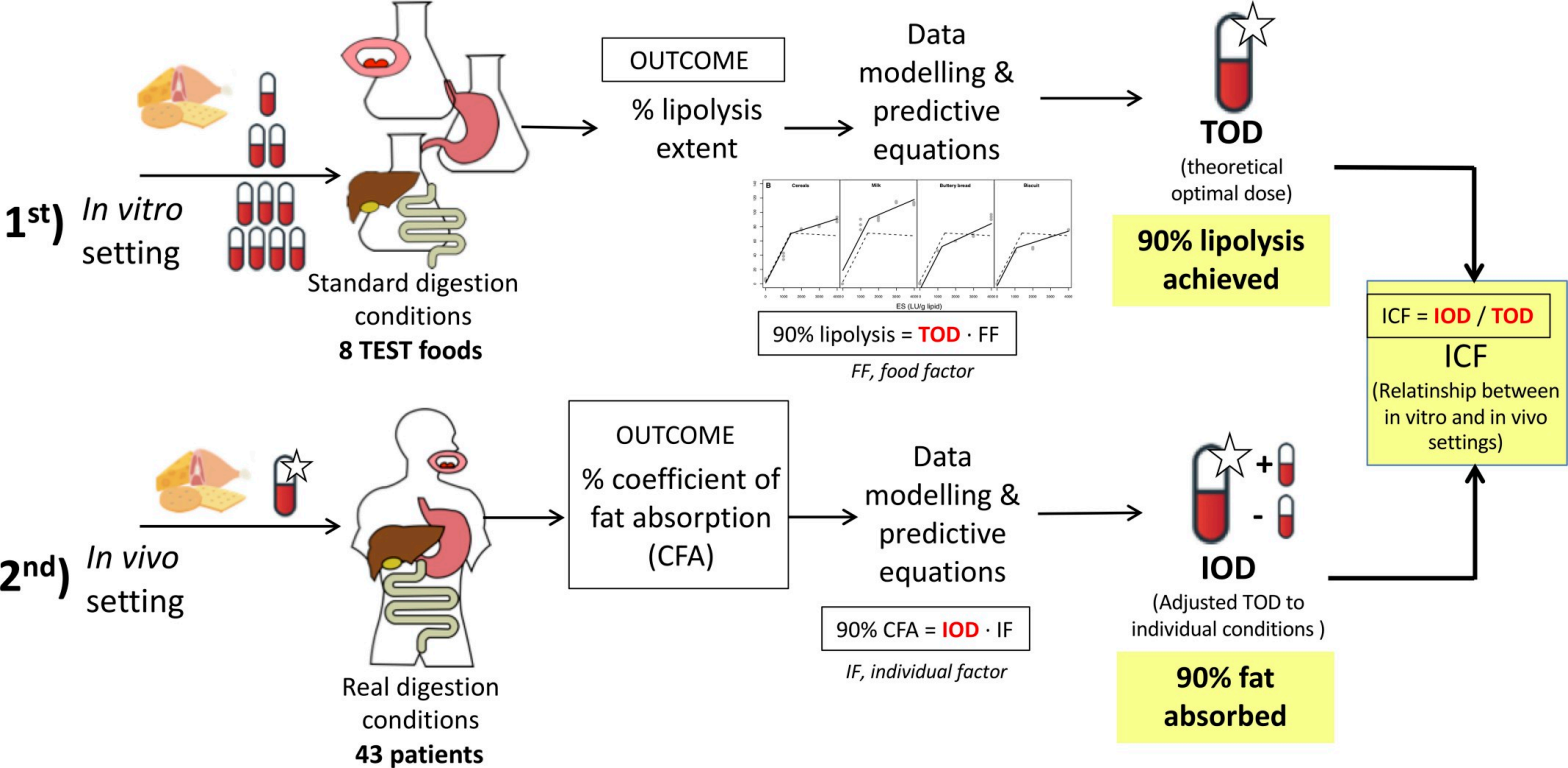
Overview of the combined *in vitro* and *in vivo* approach to validate a new evidence-based predictive model to optimally adjust the dose of enzymatic supplements used in PERT, adapted to food and patient characteristics. First a selection of 8 meals were digested *in vitro* under standard CF conditions, and the % of lipolysis was obtained for the different doses of the enzymatic supplement. Modelling of the % lipolysis extent allowed predicting the theoretical optimal dose for each meal (TOD), which took into account the food characteristics. Second, in the *in vivo* study the same 8 meals were digested *in vivo* under real conditions along with their TODs, and the % of fat absorption was measured in all the participating patients. Modelling of the coefficient of fat absorption (CFA) allowed to obtain how different TOD should have been in order to achieve a 90% of CFA (the equivalent outcome to the *in vitro* setting). In authors’ criterion, the individual correction factor (ICF) allowed for quantifying the relation between the *in vitro* and the *in vivo* settings. *TOD*, *theoretical optimal dose; IOD*, *individual optimal dose; ICF*, *individual correction factor*.

We carried out a multicentre, prospective, interventional pilot study. All the patients followed a fixed, predefined study diet composed by the previously *in vitro* digested foods, in combination with a fixed predefined dose of enzymatic supplements used in PERT based on the TOD obtained in the *in vitro* digestion studies (Fig 1). The enzymatic supplements consisted of enteric-coated microspheres of porcine-origin pancreatin, including amylase, protease and mainly lipase.

Patients were provided with the study protocol, which included a detailed instruction handbook to perform the study and as a weighted food record. Structured as a 3-day diary, the study protocol showed pictograms with the exact amount of each food item to be registered by the patient or the caregiver and the corresponding dose of enzymes for each meal. When a higher or a lower amount of the indicated portion for each food was taken, the patient had to indicate it, but the indicated dose of the enzymatic supplement was maintained. Also questions about adherence to PERT and test diet were asked. The only extra foods permitted between test meals were fat-free food, such as fruit or jelly, which did not require the intake of enzymatic supplement. The researchers provided a pillbox with different compartments containing the predefined TOD of the enzymatic supplement labelled for each test meal. When TODs were in between the regular capsule sizes, half of the content of a capsule was emptied in order to meet the recommended LU. A form was asked to be filled at the end of each day reporting the number of bowel movements, deposition time, colour and Bristol stool score. Patients were asked to collect faeces corresponding to the ingestion of the study menu: this was made possible by using colorimetric markers at the start and at the end of the study diet. The colorimetric markers (blue and red) were also included in the pillbox given to the participants.

At the study visit, patients’ individual characteristics were collected as study variables: age, gender, intake of proton pump inhibitor, BMI z-score, and CFTR mutations, defined as Class I, Class II and Class III [15]. The study protocol was approved by the ethical committee of each centre: Hospital Universitari i Politècnic La Fe (Valencia), Hospital Universitario Ramón y Cajal (Madrid), Ospedale Maggiore Policlinico (Milan), Universitair Ziekenhuis Leuven Hospital (Leuven) and Erasmus MC Sophia’s Children Hospital (Rotterdam). All parents and patients were informed about the purpose and ultimate aim of the study and gave written informed consent.

#### The test diet and study doses

**Fig 2:**
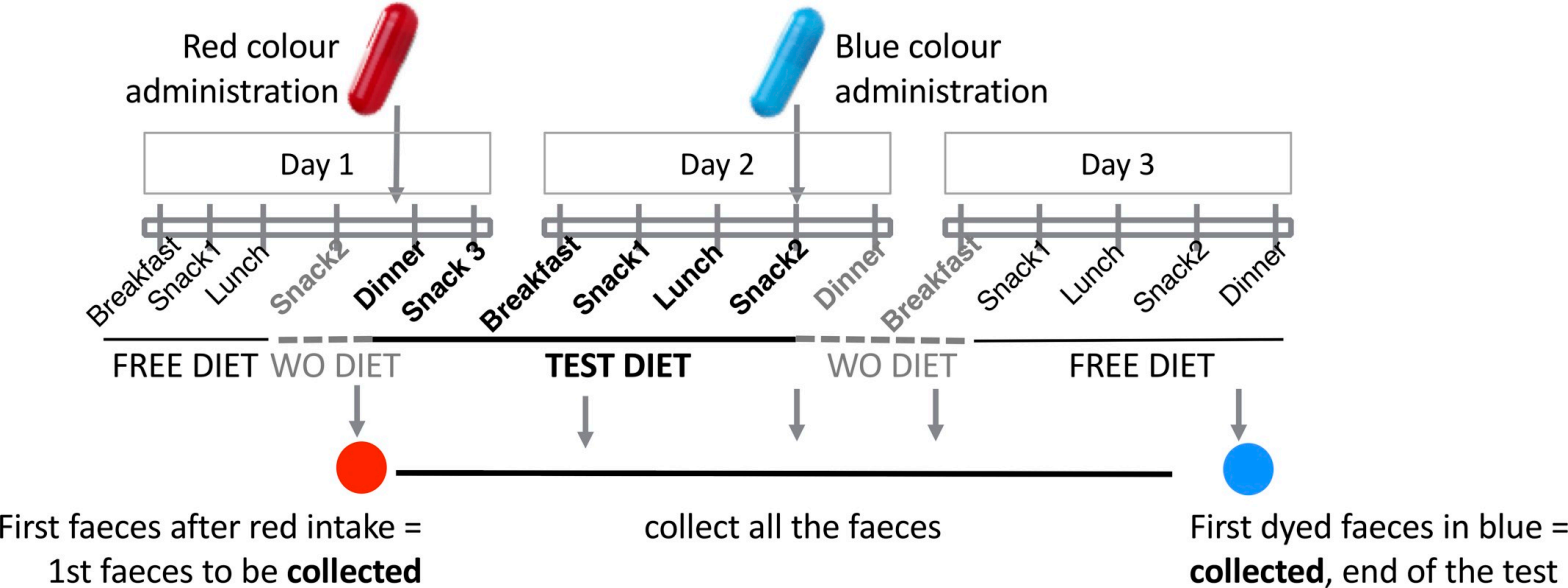
Overview of the study. The study started at day 1 afternoon with a washout diet including a fixed dose of the enzymatic supplement (TOD) (snack 2) and the administration of the red marker. From then on, and during day 2 patients followed the test diet with the fixed dose of the enzymatic supplement (TOD), until the afternoon snack on day 2, that was taken in combination with the blue markers. The dinner of day 2 and the breakfast of day 3 conformed the washout diet after the test menu, also with a fixed dose of the enzymatic supplement (TOD). Faecal samples had to be collected from the moment of the red colour intake until the first blue-dyed deposition was found. For the faecal analysis, researchers selected the samples comprised from the 1^st^ red deposition to the 1^st^ blue. *WO*, *washout*.

The study period included a test diet composed by 6 meals distributed in 24 hours, and also a washout diet period before and after the test diet itself (3 meals) to minimise the impact of the regular and uncontrolled normal diets of the participants in the faecal analysis. All the meals included in the test diet had been previously digested *in vitro* and the corresponding TODs were estimated considering the most unfavourable intestinal conditions of pH 6 and bile salts concentration of 1 mM according to previous literature [16–18]. Fig 2 provides the overview of the study timeline per patient.

In the design of the meals the following considerations were taken into account for the feasibility and to avoid country-related potential bias: meals had to fit all the nutritional habits according to previous results of our group [19], international brands had to be used in order to achieve the same nutritional composition and easy cooking techniques were to be used, so that the assumption that the TOD was maintained in the foods used in all the countries could be made. To calculate the nutrient profile of the test meals, the nutritional composition databases of EuroFIR and Nubel were upgraded including the nutrient information directly from the specific test food labelling. Four energy levels were proposed so that patients of all ages could participate: 1622, 1832, 2194 and 2573 kcal/24h. In all of them, the macronutrient distribution was approximately 40% lipids, 40% carbohydrates and 20% protein, according to the nutrition guidelines [1]. In each level, the foods were the same, but the amount varied and TOD dose was adapted proportionally (Table 1). Therefore, the dose of the enzymatic supplement in LU/g of fat was the same for all the participants, regardless the amount of food they had. Families and patients were allowed to choose the level of energy together with the dietitian that most appropriately fitted the appetite and habits of the participant. Participants were strictly instructed by the investigators to purchase the indicated brands for each product.

**Table 1:** Theoretical optimal doses of enzymatic supplement (TOD) expressed in lipase units per gram of fat (LU/g fat) for a selection of eight foods previously determined by means of an in vitro digestion model. Combination of the foods conformed the meals used in the study protocol in order to assess the efficacy of the TOD assigned to each food.

Test meal	Fat content (g/ 100g)	Theoretical optimal dose of enzymes (TOD) (LU/ g fat)	Portion size (g) per test diet level (L^a^)	Fixed dose of enzymatic supplement based on TOD (total LU)
Salad with olive oil	10	1613	L1: 70	11291
			L2: 100	16130
			L3: 100	16130
			L4: 150	24195
Ham & cheese Pizza	8.3	1375	L1: 170	19401
			L2: 220	25107
			L3: 220	25107
			L4: 250	28531
Yoghurt	10	1240	L1: 125	15500
			L2: 125	15500
			L3: 125	15500
			L4: 125	15500
Ham & cheese Sandwich	10.2	1660	L1: 110	18625
			L2: 130	22011
			L3: 140	23704
			L4: 140	23704
Milk	3.6	1480	L1: 200	10656
			L2: 200	10656
			L3: 250	13320
			L4: 250	13320
Buttery bread	28.3	3400	L1: 40	38488
			L2: 40	38488
			L3: 40	38488
			L4: 40	38488
Cereal	4	4720	L1: 30	5664
			L2: 30	5664
			L3: 50	9440
			L4: 50	9440
Biscuit	25.9	6130	L1: 11	17464
			L2: 22	34928
			L3: 33	52293
			L4: 44	69857

^a^ L1, level of energy 1, 1622 kcal/24h; L2, level of energy 2, 1832 kcal/24h; L3, level of energy 3, 2194 kcal/24h; L4, level of energy 4, 2573 kcal/24h

#### Faecal collection and analysis

Detailed instructions were given to minimise the error in the CFA calculations. From the moment of the red marker intake, all the faeces had to be collected individually into separate plastic bags. Fecotainers (AT Medical BV, The Netherlands) were provided to the patients to ease the collection. This process was repeated until the blue colour was identified in the faeces.

The faeces were shipped from all the participating centres to Instituto de Investigación Sanitaria La Fe (Valencia, Spain) for central analysis. Homogenisation of all samples from each patient was carefully carried out, including only the faeces corresponding to the test menu: the samples that were collected before the appearance of red-dyed faeces were discarded. Samples considered for the analysis comprised those collected between the 1^st^ red and the 1^st^ blue-dyed, both included. Therefore, the quantification of fat in faeces considered for the CFA calculation was as accurate as possible, given that only fat corresponding to the test diet was considered. Then household stirrers 750 W were used to mix the faeces contained in the original recipients until complete homogenisation was observed (5 minutes per sample approx., depending on the consistency and volume). This faecal homogenisation method was chosen because of the lack of a standardised method in the literature. Previous studies focused on faecal analyses did not describe the protocol for homogenisation in detail, or did not make it available [20–23].

Careful cleaning of the equipment was performed between samples. Then the homogenised sample was aliquoted in 10 g recipients and kept under freezing conditions (-20°C).

Quantitative faecal fat analyses were carried out by the infrared spectroscopy technique, the gold standard to perform fat in faeces analyses at the Reference Laboratory (Barcelona, Spain). Random selected samples (n = 10) were used to assess the homogenisation protocol developed. Test results were highly reproducible (CV <15%) between two different aliquots of the same homogenised sample.

The CFA was calculated as the percentage of fat excreted in faeces (considering the faeces belonging to the test diet) compared to the fat in the test diet. This parameter is considered equivalent to the % lipolysis extent used in the *in vitro* setting. The transit time was calculated as the time between the ingestion of the red capsule and the moment the first red faeces appeared, despite the fact that this method of calculation may have only allowed for an estimated result.

The Bristol Stool Scale was used for patients to report the faecal consistency [24]. This scale is used to assess gastrointestinal complications, and ranges from 1 to 7, where 1 is extreme constipation and 7 extreme diarrhoea, and the optimal types are 3–4.

### Calculations and statistical analyses

Sample size calculation was performed assuming that differences in undigested fat between in-vitro and in-vivo digestions would range between 0 g and 18 g, with a mean value of 9 g and a standard deviation of 3 g (according to a retrospective review of clinical data from patients from one of the participating centres [7]). With these values, 41 individuals would be needed to achieve a precision of ±1 g of fat (95% CI length of 2) in the determination of the differences in undigested fat between in-vitro and in-vivo values. Assuming a drop-out of 20% of the recruited patients, the sample size was estimated to be 50 subjects.

For the descriptive analysis, data were summarised using mean (standard deviation) or median (1st, 3^rd^ Q.) in the case of continuous variables and with relative and absolute frequencies in the case of categorical variables.

A beta regression model was applied to study the association between the CFA and the selected study variables: age, dose of the enzymatic supplement (i.e. TOD), use of proton pump inhibitor (PPI) and transit time.

The results of the beta regression model can be interpreted with the exp(estimate) and the CI 95%. An exp(estimate) >1 means that the variable is positively associated with the response variable and the higher the value, the higher the effect. Complementarily, the confidence intervals that do not contain 1 are those significantly associated with the response variable.

This model was applied to calculate the optimal dose of enzymes for each patient (individual optimal dose, IOD) to reach a target of 90% lipolysis extent (i.e., CFA), according to the individual patient characteristics by means of the equation of the model (Eq 1).

$$g(\%CFA)=\beta_{0}+(\beta_{1}\cdot transit\;time)+(\beta_{2}\cdot IOD)+(\beta_{3}\cdot age)+(\beta_{4}\cdot PPI\;intake)$$ (1)

where g (·) is the logit function of the beta regression, IOD is the individual optimal dose of enzymes and PPI is proton pump inhibitors. *β*_*i*_,*i* = 0,…,4 are the model parameters.

Therefore, to calculate the individual optimal dose (IOD) for each patient to reach the clinical target of 90% CFA, this equation should be solved (Eq 2):

$$IOD=\frac{g(90{\%}_{clinical\;target\;CFA})-\beta_{0}-(\beta_{1}\cdot transit\;time)-(\beta_{3}\cdot age)-(\beta_{4}\cdot PPI\;intake)}{\beta_{2}}$$ (2)

Then, the relationship between the individual dose of enzymes and the actual TOD taken by the patient in the study conformed the individual correction factor. The individual correction factor is thus the ratio that reflects the relationship between the *in vitro* and the *in vivo* settings of the digestion (**Eq 3**). This ratio, thus, expresses how different the TOD has to be, either higher or lower, than the one predicted *in vitro*, encompassing the individual characteristics that differ from those standard characteristics simulated *in vitro*.

$$ICF=\frac{IOD}{TOD}$$ (3)

Where ICF is the individual correction factor; IOD is the in vivo optimal dose obtained by Eq 1 in which patients characteristics were considered, in order to reach a 90% of CFA; and TOD is the theoretical optimal dose of enzymes obtained in vitro, which was actually taken by the participants during the study.

A mixed ordinal regression model was carried out to study the association between CFA and the response Bristol Stool Scale due to the ordinal nature of the response (type 1 trough 7, not a continuous scale). Furthermore, because the faeces samples from the same patient are more likely to have a similar Bristol Stool Scale number than those from other patients, the model was extended with the "Patient" variable as a random effect with a random intercept to correct for the non-independence of the data.

Patients’ compliance with the study protocol was defined as intake of fat and dose of enzymatic supplement as compared to the indicated amount (%).

All the analyses were performed using R software (version 3.3.3), and lme4 (version 1.1–12), nlme (version 3.1–131), ordinal (version 2016.6–28), betareg (version 3.1–0), clickR (version 0.3.35) packages. A p-value below 0.05 was considered statistically significant.

## Results

A total of 54 patients were enrolled in the study between April and June 2017: 9 from Madrid, 12 from Valencia, 5 from Milan, 13 from Leuven and 9 from Rotterdam. Eleven participants dropped out due to stress for defecating in bags (n = 5), because of severe infections that were not related to the study (n = 2) and antibiotic use (n = 2), so the final number of participants was 43.

### Patients characteristics and descriptive results

Table 2 shows the patient characteristics of the study population and the descriptive results of the pilot study. Median age, BMI z-score and transit time were 9.4 years, -0.02 SD and 28.5h respectively. The median dose of enzymatic supplement was around 2599 LU/g fat, according to the indications in the protocol for the participants. Compliance with the test diet and the test dose of enzymatic supplement (TOD) were 98.4 and 100% respectively. None of the collected variables presented statistical differences among centres. None of the recruited patients reported adverse events related to the study nor symptoms or complaints with the exception of one patient who experienced an episode of acute diarrhoea. None of the patients were taking laxatives at the time of the study nor had previous gut resection.

**Table 2:** Patients’ characteristics and results of the pilot study in which an in vitro digestion method was set-up to assess the optimal dose of pancreatic enzyme replacement therapy according to food characteristics.

	Madrid (n = 9)	Valencia (n = 10)	Milan (n = 5)	Leuven (n = 12)	Rotterdam (n = 7)	Total (n = 43)
Age (years) *Median (1*^*st*^, *3*^*rd*^ *Q*.*)*	12.1 (9.3,14.5)	7.5 (6.9,16.3)	8.2 (6.2,11.6)	7.4 (7.2, 8.5)	10.1 (9.6, 12.9)	9.4 (6.8, 13.8)
Male, *n (%)*	6 (67)	5 (50)	2 (40)	7 (58.3)	3 (43)	23 (55)
BMI z-score *Median (1*^*st*^, *3*^*rd*^ *Q*.*)*	0.01 (-0.08, 0.07)	-0.24 (-1, 0.41)	-0.26 (-0.94, 0.58)	0.08 (-0.5, 0.3)	-0.02 (-0.2, 0.66)	-0.02 (-0.64, 0.42)
CFA (%) *Median (1*^*st*^, *3*^*rd*^ *Q*.*)*	91.2 (84.7, 93.9)	88.8 (84.9, 92.8)	94.1 (84.7, 95.2)	88.1 (79.7,91.47)	89.9 (87.5, 94.3)	90.0 (83.7, 94.4)
Total fat in faeces (g) *Median (1*^*st*^, *3*^*rd*^ *Q*.*)*	7.8 (6.7, 9.5)	8.1 (4.85, 10.62)	5.9 (3.5, 9.6)	10.35 (7.2, 15.8)	9.45 (5.1, 11.1)	8.4 (4.8, 12.3)
Transit time (h) *Mean (SD)*	24.2 (15.1)	25.9 (12.9)	30.4 (19.2)	30.0 (10.7)	32.1 (13.2)	28.5 (15.1)
Proton Pump Inhibitors (PPI), *n (%)*	3 (33.3)	4 (40)	1 (20)	4 (33.3)	2 (28.6)	14 (33)
TOD (LU/g fat) *Median (1*^*st*^, *3*^*rd*^ *Q*.*)*	2271 (2243, 2540)	2802 (2315, 2866)	2509 (2000, 2579)	2706 (2557, 2786)	2624 (2602, 2977)	2599 (2310, 2817)
Compliance with test diet (%) *Median (1*^*st*^, *3*^*rd*^ *Q*.*)*	108.7 (97.0, 112.2)	94.7 (87.0, 105.5)	99.9 (93.2, 100.0)	100.1 (92.5, 106.4)	97.0 (88.8, 105.3)	98.4 (92.5, 108.3)
Compliance TOD (%) *Median (1*^*st*^, *3*^*rd*^ *Q*.*)*	100 (89.4, 100)	94.11 (84.7, 100)	85.5 (62.7, 100)	100 (100, 103.4)	100 (94.4, 100)	100 (92.5, 100)

Compliance exceeding 100% means that amount of fat or enzymes was higher than the indicated in the protocol

#### The coefficient of fat absorption CFA

**Fig 3:**
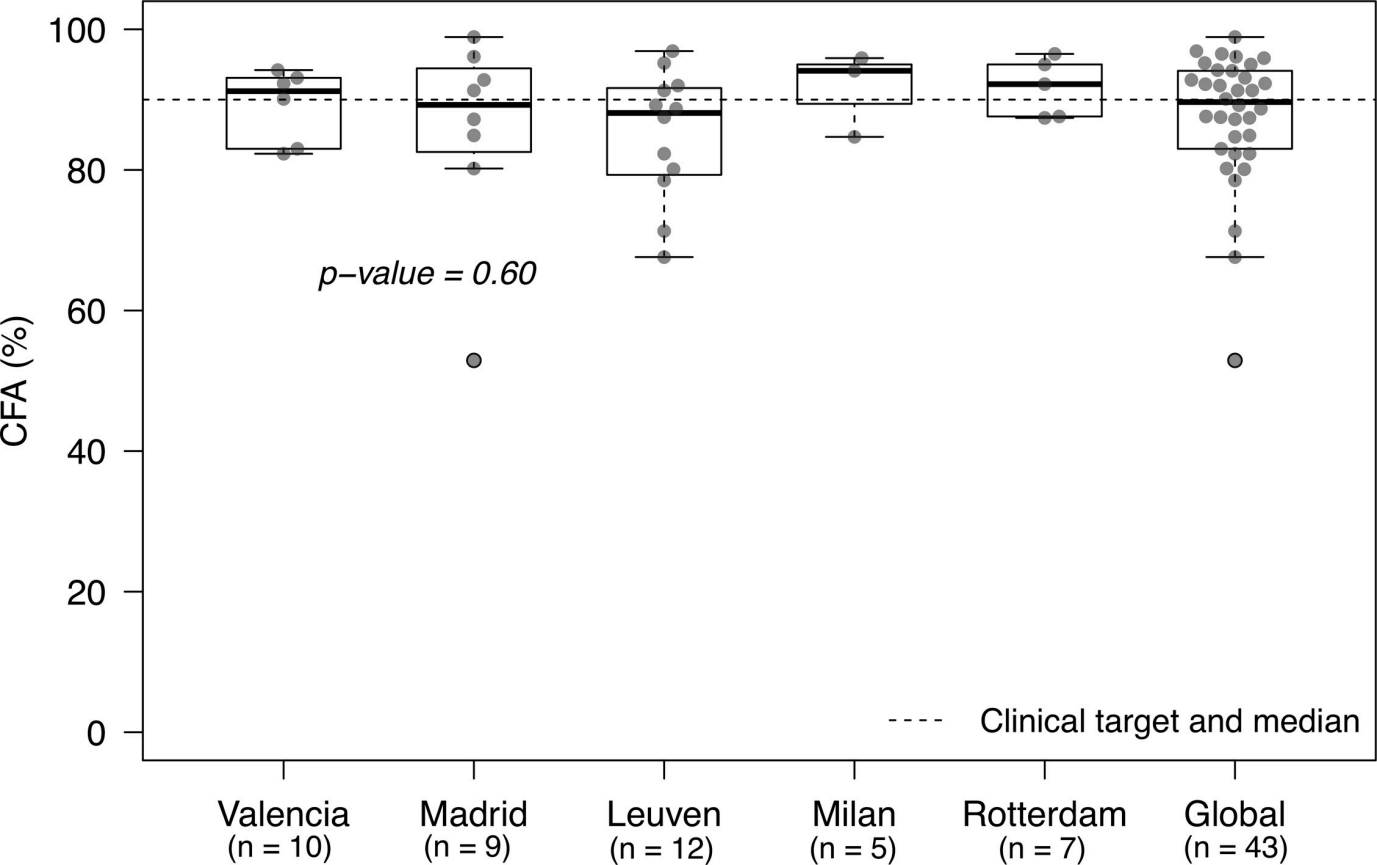
Results of the coefficient of fat absorption (CFA) obtained in the participating centres. The horizontal dotted bar represents the median CFA of the total population, coinciding with the clinical target of 90%. Boxplots represent the median and the 1^st^ and 3^rd^ quartiles in all the centres.

Overall, median CFA value was 90.0% and analysis of data for individual centre revealed the same distribution of CFA values. When analysing results of each centre individually the same distribution was found in all (Fig 3), which ranged from 83.7% (1^st^ quartile) to 94.4% (3^rd^ quartile) and no significant differences in CFA between centres (p = 0.60). There were three outliers: one in Madrid (CFA 53%) due to the inaccurate faecal collection; and two in Leuven, one developed diarrhoea with reported Bristol stool scale of 7 and transit time of 12 h (CFA 61%) and the other due to the low adherence the protocol (CFA 68%).

#### Bristol Stool Scale and CFA

**Fig 4:**
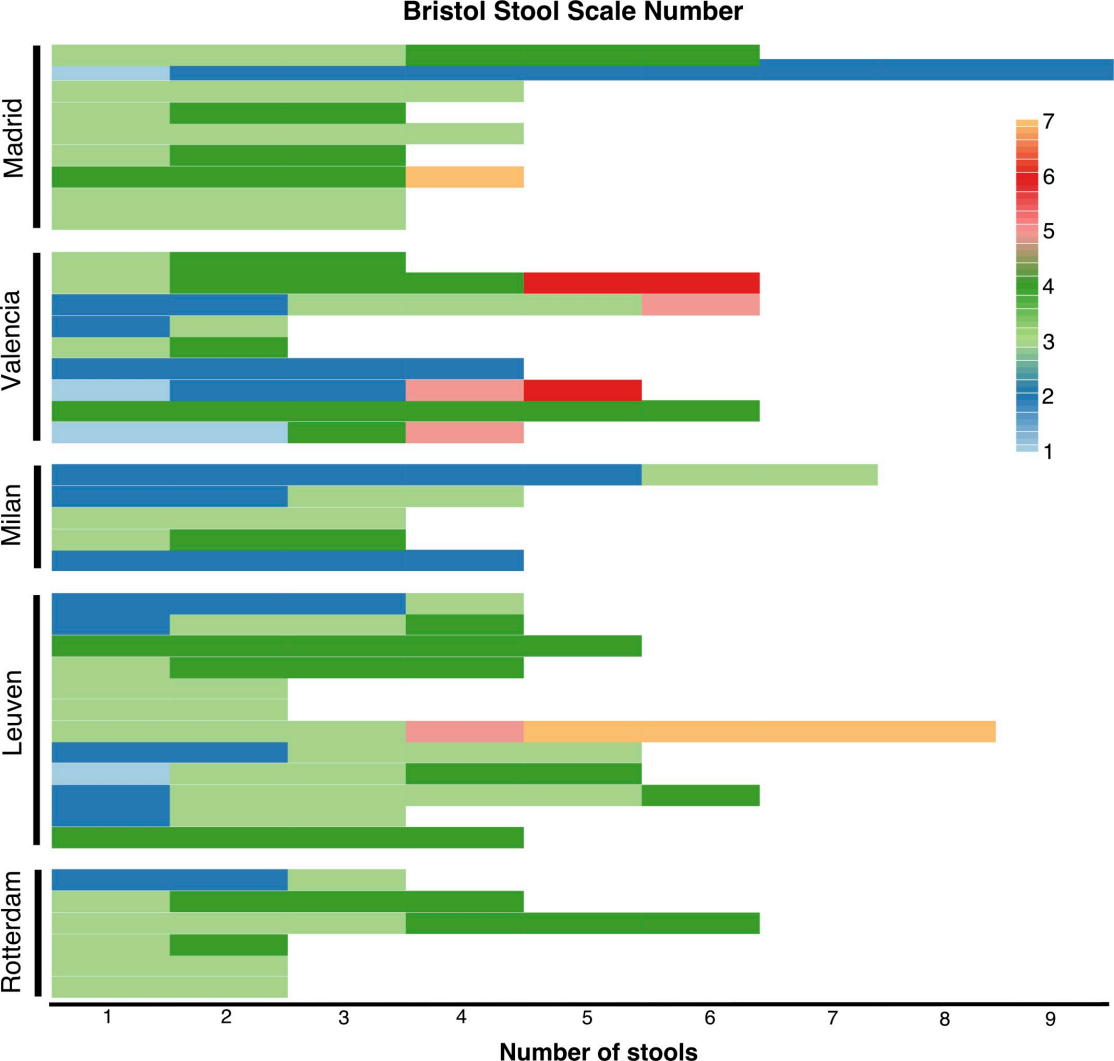
Representation of number of faecal depositions per patient and centre and the Bristol stool scale type assigned to each individual deposition by the patient or caregiver (one patient per row). Faecal samples correspond to the study period, from the red to the blue coloured deposition.

Most of the faecal samples were identified by the participants as Bristol stool scale types 3 and 4 (normal) and 2 (constipation) as shown in Fig 4. No association was found between CFA and Bristol Stool Scale number (p >0.05).

### A beta regression model to predict the individual correction factor

Table 3 shows the parameters of the beta regression model including the variables that were selected. The variables type of CFTR mutations and BMI z-score were not included in the model as mutations were severe in all the patients and the BMI z-score did not show any effect in alternative models explored previously. As for the selected variables, the CFA was significantly associated with the transit time (p = 0.007): the longer the transit time, the higher the CFA (CI 95% [1.177, 2.797]). The CFA was not significantly associated with PPI intake, TOD and age (p = 0.09, p = 0.14 and p = 0.62 respectively).

**Table 3:** Beta regression model to assess the influence of the study variables on CFA, including the dose of enzymes (TOD) and the individual factors intake of proton pump inhibitors (PPI), age and transit time.

	(exp)Estimate	Confidence Interval CI 95%	p-value
(Intercept) (*β*_0_)	2.839	[0.223, 36.147]	0.42
TOD (*β*_2_)	0.999	[0.998, 1.000]	0.13
PPI intake (*β*_4_)	1.367	[0.885, 2.115]	0.09
Age (*β*_3_)	1.013	[0.961, 1.069]	0.62
Transit time (*β*_1_)	1.815	[1.177, 2.797]	0.007

The resulting equation (**Eq 4**) of the beta regression model was applied to predict the individual optimal dose of enzymatic supplement, which is what the dose should have been in order to achieve a 90% CFA instead of the CFA value actually obtained with the TOD. This change would have been determined by the individual patient characteristics (transit time, age and PPI intake).

g(%CFA)=1.043+(0.596·transittime)−(0.0004·IOD)+(0.013·age)+(0.312·PPIintake) (4)

where g (·) is the logit function of the beta regression, IOD is the individual optimal dose of enzymes and PPI is proton pump inhibitors.

Then Eq 3 was applied in order to determine the relationship between the TOD and the individual dose of enzymes, or the relationship between the in vitro and the in vivo setting. This relationship would have conformed the individual correction factor. The individual correction factor median was found to be 0.95 (0.92, 1.01) in all the cases, meaning that optimal dose obtained in vitro (TOD) was equivalent to the required dose in vivo (individual optimal dose).

## Discussion

Through the present study we have moved from an *in vitro* experimental set-up to an *in vivo* setting to evaluate the model for adjusting the dose of enzymatic supplements used in PERT in CF. The *in vitro* digestion study allowed for the estimation of a TOD of the enzymatic supplement for different meals and foods, and in this pilot study we have assessed the validity of this approach. We had hypothesised that when applying the TOD in vivo, specific individual corrections would be needed based on the individual subject’s characteristics. However, we found that these characteristics did not imply a modification of the dose predicted in the lab, being the relationship between both settings close to 1. In other words, our results suggest that the food characteristics are the determinants of the requirements of enzymatic supplement, and not the individual patient characteristics we have assessed.

In our clinical setting, using the TOD of enzymatic supplements, the median CFA was found to be 90%, which is slightly higher than in previous studies, and comparable to others without predefined dose [8,9]. In a similar CF-population from Valencia a mean CFA of 86% was reported [9]. In that study a high variability between patients, and within patients (differences in CFA in 3 consecutive days) along 3 visits in a year was found and attributed to the uncontrolled diet and dose of enzymes for the specific meals [9]. In contrast, in the present study with a controlled diet, we found a lower between-patients variability (84 to 94% 1^st^, 3^rd^ Q.), which suggests that the TOD had similar responses in all the participants. This is confirmed by the fact that most of the faeces were scored types 3–4 on the Bristol stool scale, which indicate the absence of gastrointestinal complications, including persistent diarrhoea and malabsorption. In addition, the patients reported no symptoms or complications so there were no gastrointestinal alterations during the study period.

With regard to the multivariate model, the equation derived from the selected parameters was supposed to be used to calculate the individual optimal dose. With the equation of the model, according to the parameters and the individual characteristics of the patients, such as age and PPIs intake, the optimal LU/g fat (individual optimal dose of enzymes) could be calculated in order to obtain the desired CFA of 90%. However, these characteristics turned out to have a very low overall effect, so the TOD and the individual optimal dose of enzymes were almost the same in all the cases (i.e. the individual correction factor was around 1 for all the patients). The positive (but not significant) effect of proton pump inhibitors has been repeatedly reported in patients with CF [25,26]. The rationale behind it is that PPI’s inhibits the acid secretion in the stomach and thus higher pH values are achieved for the intestinal digestion phase. Lipase activity is directly associated with the intestinal pH [27]. This phenomenon was clearly observed previously [15].

There are several limitations in this study. First, performing the test at home may have caused deviations from the protocol, which implied high complexity to the patients. However, in order to minimise complications, a thoughtful protocol for the participant was given in which detailed information about the type of foods and brands was specified. In addition, a questionnaire after each meal was included and finally a depositions registry had to be filled in to assess the degree of adherence, which was found to be nearly 100% despite the potential risks for deviations. Second, the number of variables included in the statistical model were limited due to the low patient number. In addition, the model relies on non-significant associations, thus its validity as a predictive model to calculate the individual correction factor in every patient is limited. Nonetheless, according to the statistical results, if more patients had been included, the same results distribution of results was to be expected as long as the protocol was strictly followed. Hence, the multivariate model would not have allowed for an individual optimal dose prediction either. Other potentially interesting variables representing patients’ individual characteristics that could have provided clearer explanation of the results could be intestinal pH and bile salts concentration, but because of the complexity and invasiveness of the methods to collect this information, we did not include these variables in the framework of the study. We indeed acknowledge that in the patients with the lower CFA results, a lower than 6 intestinal pH could be the reason for that finding. However, most of the patients had a high CFA value, and was probably the main reason for no statistical association of CFA and other study variables (apart from transit time). Another limitation is that the present study design relied on the results generated by an *in vitro* digestion model simulating CF gastrointestinal digestion. Finally, despite some assumptions or simplifications were made in this model, the CFA results obtained suggests that the real and more complex physiological conditions of *in vivo* digestion (for example, possible higher pH or bile salts concentration or residual pancreatic function) may have contributed to compensate for these possible limitations.

In contrast, the strengths of the present study rely on the robust methodology. To our knowledge, there are no studies assessing the dose of the enzymatic supplement and CFA in the context of such an accurate and detailed protocol for faecal collection, homogenisation of faeces samples, analysis and CFA calculation. In our study for the first time CFA was estimated while on a fixed diet and a fixed dose of enzymes, and in addition using an evidence-based approach.

Overall, when strictly following the protocol (including test menu, TODs and faecal collection) and in the absence of adverse events interfering with fat digestion and/or absorption, TODs were precise enough to obtain good CFA values and no other patient-related factors with a relevant influence on the individual correction factor could be postulated.

Our findings may open a new perspective on the treatment of pancreatic insufficiency in CF. Currently patients with CF have to decide the dose of enzymatic supplement to take every single time they eat. As a matter of fact, large differences in dosing criteria were previously documented between European countries, evidencing the lack of a common consensus [10]. The newly developed evidence-based method to adjust PERT could be implemented in the clinical practice to support clinicians and patients’ decision on the dose of the enzymatic supplement, possibly contributing to a more efficient nutrient digestion and absorption, and consequently, to a better nutritional status.

We showed that the digestion of fat is more dependent on the food characteristics than on the patient characteristics. Ideally, a dose tailored at each single meal could be foreseen. For this implementation, however, a supporting system would be required. In MyCyFAPP project, we have developed a mobile app including a calculation algorithm of the optimal dose of enzymatic supplements based on a database of TODs. The next step of MyCyFAPP project will assess the effects of using this app on health and quality of life-related outcomes. However, before moving to a larger study cohort for validation, this pilot study was necessary prerequisite. The results obtained seem to indicate this pilot study suggests further research in this innovative approach.

In conclusion, we have made a step ahead in the understanding of PERT dosing and we have set up the first evidence-based method to adjust the dose of the enzymatic supplement to food characteristics in CF. Although we expected that optimal dosing of the dose would be based not only on the theoretical optimal dose obtained in vitro but also by adding an individual correction factor related to the characteristics of each patient, this could not be defined. Since the in vitro predicted TODs led to the clinical target CFA results in vivo, it can be reliably used as a first approach towards further research. We should evaluate the effect on nutritional outcome as well as the overall lung function in future.

## Supporting information

S1 Table Study database. The database includes all the study variables and the response variable being the coefficient of fat absorption (CFA).
(XLSX)

S2 Table Bristol stool scale database. The database was used to represent Fig 4.
(XLSX)

S1 File Code for the statistical analyses with R software. (R)
